# *Wolbachia* Induces Epigenetic and Transcriptional Modifications in the Orthopteran *Chorthippus parallelus* (Acrididae: Gomphocerinae)

**DOI:** 10.3390/ijms27094060

**Published:** 2026-04-30

**Authors:** Patricia Jiménez-Florido, Rosario Planelló, David Buckley, José L. Bella

**Affiliations:** 1 Departamento de Biología (Genética), Facultad de Ciencias, Universidad Autónoma de Madrid, E28049 Madrid, Spain; patricia.jimenez@uam.es (P.J.-F.); david.buckley@uam.es (D.B.); 2 Centro de Investigación en Biodiversidad y Cambio Global (CIBC-UAM), Universidad Autónoma de Madrid, E28049 Madrid, Spain; 3 Molecular Entomology, Biomarkers and Environmental Stress Group, Faculty of Science, Universidad Nacional de Educación a Distancia (UNED), E28232 Las Rozas de Madrid, Spain

**Keywords:** Orthoptera, *Wolbachia*, transcriptional biomarkers, epigenetics, methylation

## Abstract

*Wolbachia* is an obligate endosymbiotic alphaproteobacterium that is widely distributed among insects. It also infects the European orthopteran *Chorthippus parallelus parallelus* (*Cpp*). In this subspecies, *Wolbachia* induces a reproductive barrier through uni- and bidirectional cytoplasmic incompatibilities. Recently, we found that it also modifies the expression of genes related to essential physiological pathways in *Cpp*. Here, we have analysed the influence of *Wolbachia* infection on the epigenetic profiles in *Cpp* gonads of infected and uninfected males and females, since they constitute *Wolbachia*’s main target. We characterised de novo nine genes related to epigenetic mechanisms and their transcriptional activity, together with global DNA methylation levels. The results indicate that *Wolbachia* influences the epigenetic mechanisms in *Cpp* mainly in females, inducing the expression of genes related to histone deacetylation and reducing the global DNA methylation percentage. This study provides the first evidence of *Wolbachia*’s ability to alter epigenetic processes in *Cpp*, increasing our understanding of this symbiotic relationship, with potential implications for the induced reproductive isolation within and between subspecies of *C. parallelus*. It also offers new insights into the molecular basis of host–symbiont biology in a group for which this information is rather scarce.

## 1. Introduction

The evolutionary success of insects has been driven by an unparalleled diversity of body plans, morphological structures, and life-history and behavioural traits. This impressive phenotypic diversity has been shaped by combining genetic and genomic novelties, new developmental pathways, the rewiring of regulatory networks and processes, and epigenetic mechanisms, among others. The epigenetic molecular mechanisms (EMMs) consisting in chemical modifications of the DNA, its associated histone proteins and RNAs affect the chromatin structure and gene expression regulation [1,2,3], and have also been revealed as important actors for the evolution of phenotypes [4]. Despite their importance, knowledge of EMMs in insects is rather limited, with most studies focusing on a small number of model species.

The three major and interacting epigenetic mechanisms are DNA methylation, chemical modifications of histones, and regulation by non-coding RNAs [3,5]. DNA methylation normally entails the addition of methyl groups to cytosine residues by DNA methyltransferases (DNMTs). In insects, cytosine methylation is overall scarce and occurs predominantly within exons of ubiquitously transcribed genes [2,5,6]. Emerging evidence shows that DNA methylation can both positively and negatively regulate gene expression depending on the genomic context and the organism under study [5,7]. Notably, variations in DNA methylation have been linked to alternative splicing events [1,2]. So, DNA methylation seems to be diverse, but its alterations influence various physiological processes and developmental pathways [3]. Moreover, differential DNA methylation has been associated with phenotypic plasticity and ecological adaptation, highlighting its importance in shaping insects’ responses to environmental changes [5,8,9]. Recently, it has also been associated with long-term evolutionary changes [10].

In addition to DNA, both the incorporation of different histone variants and their post-translational chemical modifications, such as phosphorylation, acetylation, and methylation, can also alter gene expression regulation. Among these, histone acetylation and methylation have been the most extensively studied in insects [3,5]. Histone acetylation is associated with the induction of gene expression by increasing chromatin accessibility, while their deacetylation is linked to transcriptional repression by promoting chromatin compaction [2]. These modifications are carried out by histone acetyltransferases (HATs) and histone deacetylases (HDACs), which have opposing functions [5]. Maintaining a proper balance between histone acetylation and deacetylation appears to be crucial for insect survival, as its disruption can negatively affect development and fertility, due to the alteration of hormonal pathways [11,12]. Unlike acetylation, histone methylation, regulated by histone methyltransferases (HMTs), does not lead to changes in chromatin compaction, but acts as an epigenetic mark which can mediate both the activation and repression of transcription [5]. This epigenetic modification is implied in various biological processes, including DNA repair, cell cycle regulation, stress responses and development [13].

Therefore, epigenetic processes in insects are notably complex, as they involve multiple interconnected mechanisms that form a highly integrated network for gene expression regulation [14]. EMMs are initiated in response to internal and external environmental signals [1,3], including physiochemical conditions, nutrition, predation, pathogens, symbiotic interactions, and other stressors that can trigger variable phenotypic outcomes [2,15,16].

Insects harbour a diverse range of endosymbionts, which frequently contribute to their diversification and evolutionary success. For instance, *Wolbachia*, an obligate cytoplasmic alphaproteobacterium that is widely distributed in arthropods, infects approximately 40% of species of insects [17,18]. *Wolbachia* is almost exclusively maternally inherited and induces reproductive modifications with sex-specific effects such as cytoplasmic incompatibility (CI). This is the most frequent *Wolbachia*-induced phenotype in insects, which causes infected males to leave no offspring (totally or partially) if they reproduce with uninfected females (unidirectional CI), or with those infected with different bacterial strains (bidirectional CI) [19,20,21]. This provides a selective advantage for infected females and thus promotes the presence of *Wolbachia* in the host population [17,21]. Hence, *Wolbachia* can play a significant role in its host divergence or even speciation, as it can represent a clear reproductive barrier between groups [22,23].

Although *Wolbachia* is considered to be an opportunistic reproductive parasite, it can establish mutualistic symbiotic relationships [17]. *Wolbachia* can promote host reproduction by increasing egg production [24] or boosting male egg size [25]. It can also alter host sex allocation in a temperature-dependent manner [26], enhance the resistance to various pathogens [27,28,29], and manipulate host metabolism by, for example, synthesising nutrients that may prove essential for some species [30,31], and modifying host behaviour [32,33,34]. These are examples of the different physiological processes, i.e., gene pathways, that *Wolbachia* can influence occasionally.

With the application of molecular tools, such as comparative transcriptome analyses, it has been possible to determine that *Wolbachia* can alter the transcriptional activity of hosts’ genes related to fundamental biological processes [30,35]. The influence of *Wolbachia* on gene expression suggested that this bacterium could also manipulate the epigenome of its host. In fact, *Wolbachia* rearranges chromatin during spermatogenesis in *Drosophila simulans* [36] and modifies the genomic imprint of the hemipteran *Zyginidia pullula* [37]. Furthermore, the impact of *Wolbachia* on the host’s DNA methylation has been studied [5,38,39,40], as it has been linked to CI in some species [41]. Moreover, it has been proposed that the broad-spectrum antiviral protection conferred by *Wolbachia* in insects may be under the control of the host’s methyltransferase gene [42,43]. This bacterium is also involved in the protection conferred by a first infection upon a second pathogenic exposure (i.e., immune priming), which is an emergent research topic in the field of invertebrate immunity [44].

*Wolbachia* infects the meadow grasshopper *Chorthippus parallelus* (Zetterstedt, 1821) (Acrididae: Gomphocerinae), which is also referred to as *Pseudochorthippus parallelus* by some authors (e.g., ref. [45]). This orthopteran comprises two subspecies with Palaearctic distribution, distinguished in morphology, behaviour, and other nuclear and cytoplasmic genetic traits: *C. p. erythropus* (*Cpe*), an Iberian endemism, and *C. p. parallelus* (*Cpp*), localised in the rest of Europe [46,47]. *Wolbachia* has been identified as a significative driver of reproductive isolation between both subspecies through uni- and bidirectional CI [19,48]. Previous studies have shown that *Wolbachia* increases the frequency of chiasmata and aberrant spermatids in the male germ line of this species [49] and that its prevalence varies across populations, depending on the host’s life cycle and environmental temperature—factors which potentially influence the spread of the endosymbiont and, consequently, its effects on the host’s reproductive biology in this and other species [50,51]. Furthermore, it is noteworthy that there are *Wolbachia* insertions in the genome of this grasshopper even among uninfected individuals [52]. More recently, we have also observed that *Wolbachia* infection alters the expression of genes related to the energy metabolism, the immune system and the reproduction in *Cpp* [53].

Gene expression is implicated in the evolution of genetic incompatibilities in interbreeding species [54] and, specifically, DNA methylation and histone modifications can contribute as major mechanisms behind intrinsic postzygotic isolation, i.e., the reduced viability or fertility of interspecific hybrids caused by genetic incompatibilities between diverged parental genomes [55]. That having been said, deciphering EMMs is crucial for understanding their potential role in the genetic divergence of these subspecies.

Considering the genetic diversity across *C. parallelus* subspecies, it is expected that general patterns of gene expression and cytosine methylation would naturally fluctuate among the taxa involved and their hybrids (see ref. [54] and references therein).

To further investigate the effects of *Wolbachia* on this species, we focused here on exploring the bacterium’s influence on epigenetic processes in it from a molecular perspective, given their importance in gene regulation and phenotypic variability, as evidenced in multiple organisms. Specifically, we examined the expression of nine genes associated with EMMs using real-time quantitative PCR (RT-qPCR) and evaluated the global DNA methylation levels in infected and uninfected adults. This study provides evidence that *Wolbachia* significantly affects EMMs, particularly in females, leading to increased expression of histone deacetylation genes and a reduction in overall DNA methylation levels. This integrative approach in a non-model species allowed us to explore the potential role of these mechanisms as contributors to the reproductive barriers induced by *Wolbachia*. Moreover, it gives new insights into the biology of *Wolbachia* and the intricate interactions it establishes with its host, laying the foundations for further studies in a group of organisms for which the available information on the subject is scarce.

## 2. Results

### 2.1. Characterisation of C. parallelus Genes

Nine genes involved in epigenetic processes were identified in the *C. parallelus* transcriptome (PRJNA665162) [56], which was previously annotated, ranging from histones and their H2A-like variants (*H2afy_1*, *H2A*, *His2Av*) to enzymes that were responsible for the EMMs of these nucleosomal proteins, including histone deacetylases (HDACs: *HDAC1*, *HDAC3*, *HDAC6* and *HDAC11*) and histone methyltransferases (HMTs: *SUV39H1* and *trx*). Their sequences were registered in the GenBank database (Table 1) as PV661662 (*H2afy_1*), PV661663 (*H2A*), PV661664 (*His2Av*), PV661665 (*HDAC1*), PV661666 (*HDAC3*), PV661667 (*HDAC6*), PV661668 (*HDAC11*), PV661669 (*SUV39H1*) and PV661670 (*trx*).

**Figure 1:**
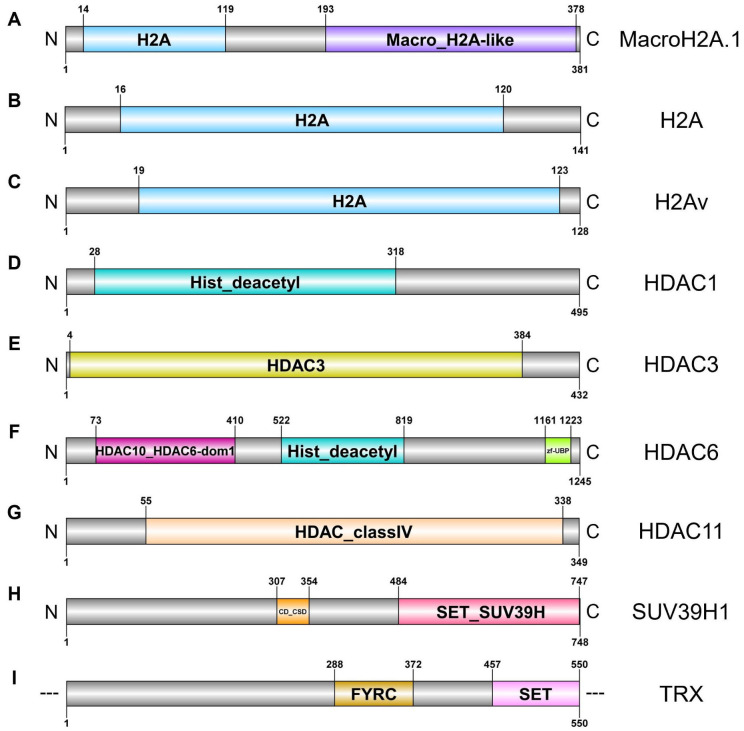
Epigenetic processes proteins identified from the de novo transcriptome of *C. parallelus*. Diagram of proteins identified as putative mRNAs, and their conserved domains: (**A**) MacroH2A.1, (**B**) H2A, (**C**) H2Av, (**D**) HDAC1, (**E**) HDAC3, (**F**) HDAC6, (**G**) HDAC11, (**H**) SUV39H1 and (**I**) TRX. H2A: Histone 2A domain, Macro_H2A-like: macrodomain, macroH2A-like family; Hist_deacetyl: Histone deacetylase domain, HDAC3: Histone deacetylase 3 (HDAC3) domain, HDAC10_HDAC6-dom1: Histone deacetylase 6, domain1 and histone deacetylase 10, zf-UBP: Zn-finger in ubiquitin-hydrolases and other protein domain, HDAC_classIV: Histone deacetylase class IV also known as histone deacetylase 11 domain, CD_CSD: CHROMO (CHRomatin Organisation Modifier) domains and chromo shadow domains, SET_SUV39H: SET domain (including pre-SET and post-SET domains) found in suppressor of variegation 3–9 homologs, SUV39H1, SUV39H2 and similar proteins, FYRC: F/Y rich C-terminus domain, and SET: SET (Su(var)3–9, Enhancer-of-zeste, Trithorax) domain. Designed using DOG 2.0.1 software [58].

A systematic search within the previously cited *C. parallelus* transcriptome provided sequences with open reading frames (ORFs) for their corresponding proteins. Eight sequences with complete ORF (*H2afy_1*, *H2A*, *His2Av*, *HDAC1*, *HDAC3*, *HDAC6*, *HDAC11* and *SUV39H1*) and one with incomplete ORF (*trx*) were obtained. The relevant domains of each ORF are shown in Figure 1.

#### 2.1.1. Histones H2A Genes

The three genes characterised presented a sequence in the ORF that encoded a protein domain of the H2A superfamily: concretely, a specific H2A domain that was characteristic of histone H2A (Figure 1A–C).

The complete ORF of *H2afy_1* (core histone macro-H2A.1 or H2A Histone Family, Member Y) was 1146 base pairs (bp) in length and encoded a 381 amino acids (aa) protein, sharing 96% identity with the orthopterans *Schistocerca americana* and *Schistocerca cancellata*. This protein comprises a H2A domain (aa: 14–119; length: 106 aa) with a H2A signature: ANVTFPV (aa: 19–25), and a macro domain (aa: 193–378; length: 186) that was representative of proteins that can bind to DNA. The ubiquitination sites were at Lys115, Lys116 and Lys118 [57]. Glycines (Gly237 and Gly330) were required for PAR (poly (ADP-ribose)) binding (Figure 1A).

The complete ORF of *H2A* (histone H2A) was 426 bp long and it contained 141 aa. This protein comprises a histone H2A domain (aa: 16–120; length: 105 aa) with a H2A signature: AGLQFPV (aa: 21–27) (Figure 1B). It shared 98% identity with *S. americana* and 97% with *S. nitens*.

The complete ORF of the histone H2A variant (*His2Av*) was 387 bp in length and coded for a protein with 128 residues; it contained the characteristic H2A superfamily domain (aa: 19–123; length: 105 aa) with a H2A signature: AGLQFPV (aa: 24–30) (Figure 1C). It shared 100% identity with the dipteran *Nasonia vitripennis* and the hemipteran *Nilaparvata lugens*.

#### 2.1.2. Histone Deacetylases (HDACs) Genes

The four genes characterised here presented a sequence in their ORF that encoded a protein domain of the Arginase_HDAC superfamily, which is representative of the HDACs enzymes. However, each of the genes encoded a protein with a different specific domain within this superfamily (Figure 1D–G).

The ORF of *HDAC1* (histone deacetylase 1) was 1488 bp long and encoded a putative protein with 495 aa. The protein presented a specific Hist_deacetyl domain (aa: 28–318; length: 291 aa), and a HDCA1 non-specific domain (aa: 7–374; length 368 aa). It shared 99% identity with those of the orthopterans *S. gregaria* and *S. americana* (Figure 1D).

The complete ORF of *HDAC3* (histone deacetylase 3) was 1299 bp in length and it contained 432 residues with a HDAC3-specific domain (aa: 4–384; length: 381 aa) (Figure 1E). This protein shared 99% identity with those of the orthopteran *S. americana* and 95% with the termite *Zootermopsis nevadensis*.

The complete ORF of *HDAC6* (histone deacetylase 6) was 3738 bp long and it contained 1245 aa. The protein showed three distinctive specific domains: HDAC10_HDAC6-dom1 (Arginase_HDAC superfamily, characteristic of class IIb HDACs, aa: 73–410; length: 338 aa), Hist_deacetyl (Arginase_HDAC superfamily, aa 522–819; length 298 aa), and zf-UB-specific domain (zf-UBP zinc finger superfamily, aa: 1161–1223; length: 63 aa) (Figure 1F). The protein shared 91% identity with those of the orthopterans *S. nitens* and *S. cancellata*.

The complete ORF of the *HDAC11* (histone deacetylase 11) contained 1050 bp; it encoded a 349 aa protein with the specific domain HDAC_classIV, characteristic of class IV HDACs (aa: 55–338; length: 284 aa) (Figure 1G). It shared 97% identity with several *Schistocerca* species, like *S. cancellata*, *S. americana* and *S. nitens*.

#### 2.1.3. Histone Methyltransferases (HMTs) Genes

Two genes coding for HMTs enzymes were characterised (Figure 1H,I).

The complete ORF of *SUV49H1* (histone-lysine N-methyltransferase SUV39H1) was 2247 bp in length and encoded a putative 748 aa protein. The protein had two specific domains: CD_CSD (CHROMO (CHRromatin Organisation Modifier) domains and chromo shadow domain; aa: 307–354; length: 48 aa) and SET_SUV39H (SET domain (including pre-SET and post-SET domains); aa: 484–747; length: 264 aa) (Figure 1H). The protein shared 84% identity with those from *S. gregaria*, *S. cancellata* and *S. piceifrons*.

Finally, the incomplete ORF of *trx* (histone-lysine N-methyltransferase trithorax) was 1650 bp long and encoded a protein with 550 aa. The structural analysis of the protein revealed two specific domains: FYRC (F/Y rich C-terminus, normally found in the trithorax family proteins; aa: 288–372; length: 85 aa) and SET (Su(var)3–9, Enhancer-of-zeste, Trithorax) domain (aa: 457–550; length: 94 aa) (Figure 1I). This protein shared 79% identity with *S. americana* and *S. piceifrons*.

### 2.2. Effects of Wolbachia on Genes Related to EMMs in Cpp

Interestingly, *Wolbachia*-dependent alterations in the expression profiles of epigenetic mechanism-related genes were observed among groups. From a general perspective, an induction of gene expression was seen under *Wolbachia* infection; however, most of the significant differences were detected in infected female gonads.

**Figure 2:**
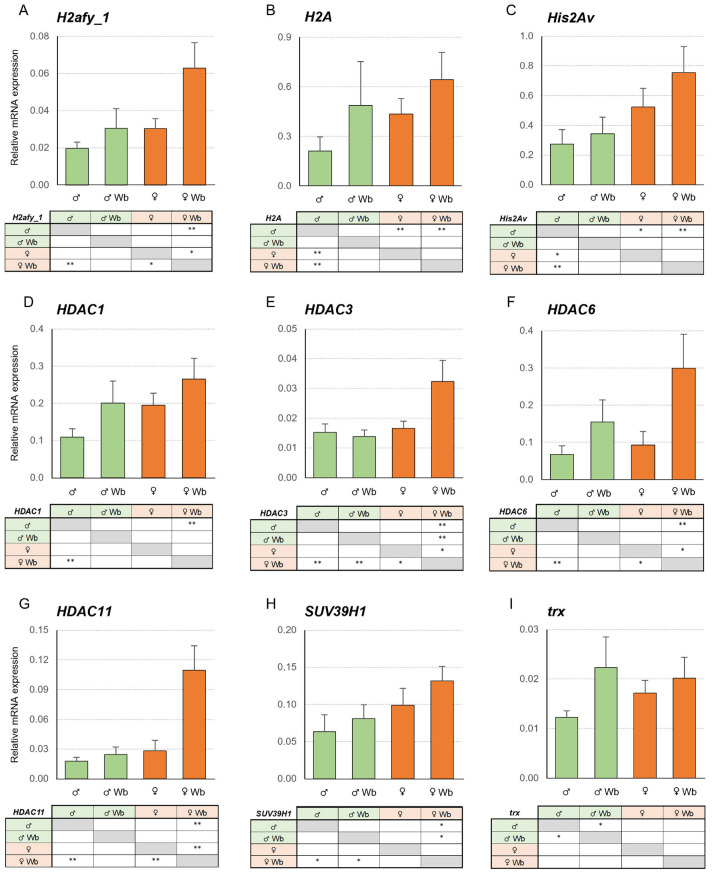
Transcriptional activity of epigenetic process-related genes in the gonads of *Wolbachia* (Wb)-infected and uninfected adult males (♂) and females (♀) *Cpp* from natural populations. Bars represent the expression patterns of the studied genes measured by RT-qPCR ± SE (males in green and females in orange). (**A**) *H2afy_1*; (**B**) *H2A*; (**C**) *His2Av*; (**D**) *HDAC1*; (**E**) *HDAC3*; (**F**) *HDAC6*; (**G**) *HDAC11*; (**H**) *SUV39H1* and (**I**) *trx*. For each experimental condition, RNA was extracted from separated individuals (n = 12 ♂; 8 ♂ Wb; 13 ♀ and 7 ♀ Wb), as indicated in the text. Significant differences between conditions: ** *p* ≤ 0.05 and * *p* ≤ 0.1.

*Wolbachia* significantly altered the expression profile of five of the nine genes analysed (Figure 2). Regarding the genes encoding histones and their H2A variants (*H2afy_1*, *H2A* and *His2Av*), significant *Wolbachia*-dependent differences were only observed in the *H2afy_1* expression profile. This gene, which encodes a variant of the major-type core histone H2A, showed significant *Wolbachia*-dependent overexpression in females, with a 3-fold increase in mean transcriptional activity in infected females relative to uninfected females (*p* = 0.076) (Figure 2A). In addition, the *H2A* and *His2Av* genes exhibited significant sex-dependent differences. On the one hand, the *H2A* gene showed significant differences between uninfected individuals, with females having a 2-fold higher expression level than males (*p* = 0.030) (Figure 2B). On the other hand, in the *His2Av* gene, uninfected females displayed 2-fold higher expression than uninfected males (*p* = 0.073) (Figure 2C).

Concerning the genes encoding histone deacetylases, the transcript levels of the *HDAC3*, *HDAC6* and *HDAC11* genes triggered a significant *Wolbachia*-dependent induction in females; up to 2-, 3- and 4-fold above uninfected females, respectively (*p* = 0.055, *p* = 0.076 and *p* = 0.035) (Figure 2E–G). *HDAC3*’s significant sex-dependent increase was also observed among infected groups, with a mean value of overexpression in infected females being up to 2-fold higher than in infected males (*p* = 0.025) (Figure 2E). Regarding the *HDAC1* activity, a similar increasing trend was observed with both *Wolbachia*- and sex-dependent, although there was no statistical significance (ns; *p* > 0.05) (Figure 2D).

In relation to histone methyltransferases, the presence of *Wolbachia* upregulated the *trx* expression in males, with a 2-fold increase in the mean value with respect to uninfected males (*p* = 0.079) (Figure 2I). This response was absent in females. In addition, the *SUV39H1* gene showed significant sex-dependent differences between infected individuals, with a 2-fold higher induction in females with respect to males (*p* = 0.077) (Figure 2H).

### 2.3. Effects of Wolbachia on the Global DNA Methylation in Cpp

**Figure 3:**
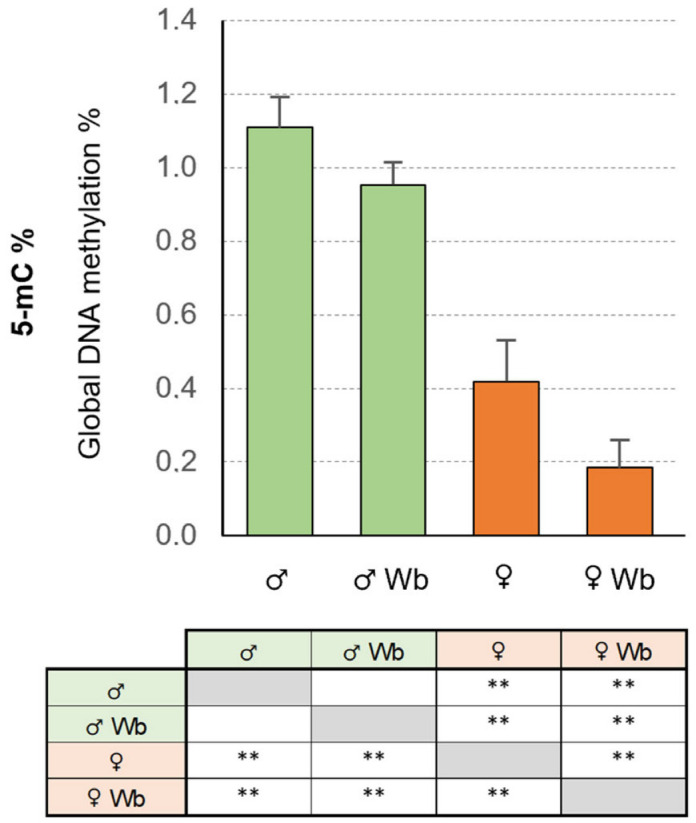
Global DNA methylation (% 5-mC) percentage in the gonads of *Wolbachia* (Wb)-infected and uninfected adult males (♂) and females (♀) *Cpp* from natural populations. Bars represent the global DNA methylation level for each of the four experimental conditions (in green males and in orange females). For each experimental condition, DNA was extracted from separated individuals (n = 10 ♂; 10 ♂ Wb; 10 ♀ and 10 ♀ Wb), as indicated in the text. Significant differences between conditions: ** *p* ≤ 0.05.

The impact of *Wolbachia* infection on the *Cpp* epigenome was evident (Figure 3), with significant *Wolbachia*-dependent differences in the 5-methylcytosine (5-mC) percentage observed exclusively in females; a similar trend was detected in males, although without statistical significance. *Wolbachia* dropped DNA methylation levels by up to 50% in infected females (*p* = 0.041). Additionally, striking sex-dependent differences in DNA methylation were found, with males showing higher methylation levels than females within the infected and uninfected groups, respectively. Within uninfected individuals, males showed a 3-fold higher 5-mC percentage than females (*p* < 0.001). Among *Wolbachia*-infected individuals, males had a 5-fold higher overall methylation level than females (*p* < 0.001).

## 3. Discussion

Given our previous findings [48,49,59,60] and related reports in other arthropod species [36,37] (see Section 1), we here investigate in an exploratory way whether *Wolbachia* affects the epigenome of *C. parallelus*. This work presents a de novo characterisation of the *C. parallelus* genes involved in various epigenetic processes, including histones, some of their variants, and enzymes that are responsible for their modification (such as histone deacetylases [HDACs] and histone methyltransferases [HMTs]) (Table 1). Using these bio-markers for epigenetic mechanisms, we have analysed and compared their transcriptional responses in uninfected/infected male and female *Cpp* adults. In the same way, we evaluated for the first time the global DNA methylation level in the gonads of *Cpp* adults, infected or not by *Wolbachia.* Overall, the effects of *Wolbachia* were evident, especially in females, both in gene expression and genome cytosine methylation percentages; sex-dependent responses were also detected in the studied parameters.

To the best of our knowledge, no previous studies have examined the impact of *Wolbachia* on the expression of any of the genes selected in this research in the species it infects. As previously mentioned, the impact of *Wolbachia* on the transcriptional activity of EMMs genes in *Cpp* is more pronounced in females than in males, which is consistent with our earlier findings on genes related to other essential physiological pathways [53].

In females, *Wolbachia* induces the expression of three of the four HDAC genes analysed (*HDAC3*, *HDAC6* and *HDAC11*). Since histone deacetylation is a common and reversible epigenetic modification that increases DNA compaction and decreases gene transcription [2], the observed overexpression of these genes in *Wolbachia*-infected females could imply that the bacterium downregulates other orthopteran genes involved in different physiological pathways, as has been documented in other species [30,61]. The induction of HDAC genes is consistent with findings in the lepidopteran *Galleria mellonella*, in which the infection with certain pathogenic bacteria increases the expression of these HDACs genes [12]. Similarly, *Wolbachia* diminishes histone acetylation in *Drosophila melanogaster* during spermiogenesis [62].

The balance between histone acetylation and deacetylation is a determining factor in insect biology, as disruptions in these processes can have broad effects on survival. Histone acetylation and deacetylation regulate gene expression, so their alteration can affect their longevity, fertility, and development by influencing key endocrine pathways [11,12]. These pathways, in turn, play a role in crucial developmental processes, such as metamorphosis, where acetylation and deacetylation have been shown to regulate pupal diapause in holometabolous insects [12,63]. Additionally, in the gonads of adult locusts, a taxonomically close species to *C. parallelus*, there is a high expression of histone-modifying enzymes, which points out their role during embryogenesis and gametogenesis [64]. Consistently, these EMMs also influence immune system responses during infection and wounding [12,44]. Hence, the impact of *Wolbachia* on these mechanisms could condition the transcriptional activity of other genes and consequently the biology of infected individuals.

Concerning the rest of the genes examined, *Wolbachia* also overexpresses the *H2afy_1* gene in females, which codes for the MacroH2A.1 histone that is related mainly to transcriptional repression [65], as occurs with histone deacetylation. In addition, *Wolbachia* induces in males the expression of *trx* gene, which encodes a histone methyltransferase that methylates the residue ‘Lys-4’ of histone H3 (H3K4me), a specific tag for epigenetic transcriptional activation [66]. HMTs, unlike HDACs, produce epigenetic modifications that can both activate and repress transcription [5]. For example, unlike *trx*, the *SUV39H1* gene encodes other histone H3 methyltransferases that cause heterochromatinization, thereby reducing gene expression [66]. In the next step, we understand that also investigating histone acetyltransferases next to the deacetylases could give a better picture and potentially reenforce if there is a change in the histone acetylation pathway.

With the limited information available, our results suggest that the endosymbiont may be altering the global transcriptional profile of *Cpp*, especially in females, by diverse mechanisms, as it differentially alters the expression of genes involved in various epigenetic modifications. This is further supported by the results obtained regarding the global methylation level of the genome of this organism. We would like to point out that in this *C. parallelus* model, the frequency of infected individuals has been shown to change significantly across geographical and temporal scales over consecutive years. This follows a temporal pattern of variation that is compatible with a negative frequency-dependent natural selection mechanism that may reflect or even be motivated by a global mechanism modifying the host physiology (host-fitness effects) [67], as presented here. Although this work focuses on the gene expression of particular genes, our findings provide the basis to develop ad hoc experimental designs to decipher these issues and the underlying mechanisms at the protein and epigenetic regulation levels.

Most of the limited studies assessing *Wolbachia*’s influence on the epigenome have focused on its effect on the global DNA methylation level, as shown in this work, and on the expression of genes encoding DNA methyltransferases (DNMTs), which are enzymes involved in such epigenetic modification [38,40,41]. However, in contrast to what has been observed in other orthopteran species [1], no genes encoding DNMTs have been found yet in the transcriptome of *C. parallelus*. As a result, their transcriptional activity could not be assessed and related to cytosine methylation in this subspecies. Likewise, the present study shows the presence of DNA methylation in this subspecies, implying the existence of a methylation toolkit behind this, even though it has not yet been identified. We want to emphasise that epigenetic mechanisms in insects, and even more so in grasshoppers, are scarcely understood, so this research contributes to an area that is still rarely explored. Regarding this, our goal is providing genome-wide data to be analysed in future, more precise approaches.

Bacterial infection swiftly modifies the DNA methylation level and, consequently, affects the activity of enzymes that are responsible for this epigenetic mechanism [68,69]. According to the results obtained, *Wolbachia* influences the overall DNA methylation level of *Cpp* gonads, although only significantly in females, which may imply that the methylation machinery is also affected. The percentage of cytosine methylation decreases by 50% in females in the presence of *Wolbachia*. Despite the CI and the cytological alterations it causes in males of *C. parallelus* [48,49,59], *Wolbachia* infection does not significantly alter their methylation profile, although it had previously been suggested that such epigenetic modification could be involved in this *Wolbachia*-induced phenotype [41]. In terms of methylation percentage, the results obtained in this study are in partial agreement with those observed in the spider *Hylyphantes graminicola*, in which *Wolbachia* infection leads to a significant reduction in the overall cytosine methylation in both females and males [38]. Contrariwise, *Wolbachia* infection leads to increased methylation in females of the parasitoid wasp *Trichogramma pretiosum* [39], and apparently also in the male gonads of *Drosophila melanogaster*, although its effect on CI is contested. Therefore, it appears that the impact of *Wolbachia* on DNA methylation is variable in its hosts depending on the species, as well as the sex and tissue being analysed, without ruling out environmental factors.

The different influence of *Wolbachia* on DNA methylation may also be associated with the type of development and metabolism of the species. This is because, even without considering *Wolbachia* infection, invertebrate species exhibit lower levels of DNA methylation than vertebrates [3]. Among insects, in which methylation is overall scarce, holometabolous species display much lower levels than hemimetabolous ones [5]. In fact, cytosine methylation patterns in insects differ from other animals, as it is preferentially targeted to a small group of ubiquitously expressed genes. Insects have higher methylation within gene bodies, especially within exons, and not in gene promoter regions, with intergenic regions and transposable elements generally being depleted of this epigenetic modification [3,5,6]. Nevertheless, the extent and genomic distribution of methylation can vary substantially among insect groups. For example, orthopteroid insects have been reported to exhibit comparatively higher levels of methylated CpG sites and of methylated repetitive elements, suggesting that epigenetic regulation through DNA methylation may be more prominent in these lineages [1].

It should be noted that significant sex-dependent differences have been detected in the expression of certain genes and in the DNA methylation level. On the one hand, the difference between uninfected males and females in the transcriptional activity of two of the three histone variants analysed (*H2A* and *His2Av*), which are more highly expressed in females, is difficult to interpret, as these are conserved proteins that are essential for nucleosome formation and the modification of chromatin conformation [3,5] but could be linked to the chromatin compaction during spermatogenesis. These large differences in DNA methylation between sexes are noteworthy. We believe that this warrants further research beyond the influence of *Wolbachia* itself, the main focus of this study. On the other hand, sex-dependent differences in infected individuals in the expression of genes coding for histone modifying enzymes (*HDAC3* and *SUV39H1*) may be caused by the differential manipulation of the transcriptional activity that produces *Wolbachia*, as discussed above. In addition, the higher rate of DNA methylation in both infected and uninfected males compared to females could result from sex-specific methylation patterns, as previously described in the mealybug *Planococcus citri* [70]. For instance, during mammalian spermatogenesis, an increase in the DNA methylation level is required for proper DNA condensation and the correct development of spermatids [71]. Although it is not straightforward to assume that DNA methylation plays an equivalent role in insects, given the considerable differences in epigenetic regulation across taxa, the limited current knowledge of their epigenetic mechanisms makes this possibility worth considering. Therefore, the observed differences could reflect the sex-specific epigenetic regulation associated with gametogenesis or other developmental processes.

The differential levels of DNA methylation and alterations in gene expression associated with *Wolbachia* infection raise the question of whether these processes are interconnected. In this regard, the relationship between DNA methylation and gene expression regulation remains contentious, particularly in insects, where the function of this epigenetic modification is still not fully understood. Previously, DNA methylation was thought to regulate gene expression, as a high percentage of 5-mC in DNA was linked to transcriptional repression, while lower methylation levels were associated with higher transcriptional activity [5]. However, more recent evidence blurred this pattern, as its effects appear to depend on the genomic context and the organism under study [7,9,10,69]. For instance, some studies reported no association between DNA methylation and gene expression [72], whereas others identified a clear relationship [5,7], linking differential methylation to phenotypic variability and ecological adaptation [8,9]. Moreover, in insects, DNA methylation has been linked to histone modifications, which also condition the chromatin structure and accessibility [1,14]. These findings suggest that DNA methylation is only one component of a broader, highly integrated network of regulatory mechanisms governing gene expression. Altogether, the existing body of knowledge underscores the complexity of these processes, which encompass different integrated mechanisms [14,72].

In this sense, further studies are required to elucidate EMMs in *C. parallelus*, particularly to deepen our understanding of their relationship with variations in gene expression profiles in the presence of *Wolbachia*. Future research incorporating whole-genome DNA methylation analyses would likely provide a more comprehensive view of the epigenetic landscape associated with the infection. Additionally, comparative transcriptomic studies across multiple species with and without *Wolbachia* infection could help determine whether consistent patterns of gene expression changes occur across hosts.

## 4. Materials and Methods

### 4.1. Sampling and Insect Population

To evaluate *Wolbachia*’s effects on gene expression, we worked with male and female adults of *Cpp* collected in Gabas (France; 42°53′60″ N 0°25′60″ W; 1020 m). These individuals are considered a pure population of the European continental subspecies *Cpp* [73]. *C. parallelus* is an annual organism, and all the samples were collected in the same year at the same time; hence, they were all adults of a very similar age. In the laboratory, gonads from both sexes were extracted for RNA isolation while *Wolbachia* infection was determined from a DNA extraction from the leg of the same individuals. A total of 40 individuals were analysed: 12 non-infected males, 8 *Wolbachia*-infected males, 13 non-infected females and 7 *Wolbachia*-infected females.

To analyse *Wolbachia*’s influence on the global DNA methylation level, genomic DNA samples from gonads were used. These samples belonged to other adult individuals of the *Cpp* subspecies, collected in the same location: Gabas (France). A total of 40 individuals were analysed: 10 non-infected males, 10 *Wolbachia*-infected males, 10 non-infected females and 10 *Wolbachia*-infected females.

In both studies, gonads were chosen as a target tissue to analyse the *Wolbachia—C. parallelus* endosymbiotic relationship due to the high bacterial load in these glands [59,60], where the bacteria presumably induce significant modifications.

### 4.2. DNA Extraction and Wolbachia Detection

The standard phenol–chloroform procedure for DNA isolation and PCR-based *Wolbachia* detection were performed as described in refs. [59,73,74]. PCR was conducted using specific primers designed to amplify a ~1400 bp fragment of the 16S rDNA bacterial genes, as already indicated in ref. [53]. Each reaction mixture consisted of 200 ng of isolated DNA, 5 μL of reaction buffer, 0.2 mM dNTPs, 1.5 mM MgCl_2_, 30 pmol of each primer, and 1 U of Taq DNA polymerase in a final total volume of 50 μL. The cycling conditions for the amplification included an initial denaturation of 3 min at 95 °C, followed by 30 cycles of 30 s at 95 °C, 1 min at 54 °C, 2 min at 72 °C, and a final extension step of 10 min at 72 °C. The positive and negative controls were set up as usual.

### 4.3. Gene Characterisation

To identify the insect genes involved in EMMs, a review of the limited existing studies examining variation in the expression of epigenetic-related genes in *Wolbachia*-infected arthropods was conducted [38,39]. This was complemented by an extensive search in databases such as UNIPROT (www.uniprot.org). To the best of our knowledge, no studies of this type have been conducted in other orthopteran species, and only a few exist for other arthropods and nematodes infected with *Wolbachia*.

Based on this search, the corresponding *C. parallelus* proteins were identified from the ORFs sequences obtained from the annotated transcriptome (PRJNA665162) [56] using the ExPASy Translate tool [75]. The amino acid sequences encoded by each ORF were analysed using the Basic Local Alignment Search Tool (BLAST^®^ v2.10.1) [76], which was available through the National Center for Biotechnology Information (NCBI; www.ncbi.nlm.nih.gov), to determine the sequence similarity with homologous proteins from other species. Additional annotation and protein characterisation were performed using the UNIPROT and KEGG (Kyoto Encyclopedia of Genes and Genomes; www.genome.jp/kegg (accessed on 24 January 2024)) databases. The functional domains of the predicted protein were visualised by generating schematic diagrams with DOG software v2.0 [58], based on domain information obtained from BLAST comparisons.

In this work, the nucleotide sequences of the following genes were identified and de novo characterised in *C. parallelus*: *H2afy_1*, *H2A*, *His2Av*, *HDAC1*, *HDAC3*, *HDAC6*, *HDAC11*, *SUV39H1*, and *trx*. No additional histone variants, histone acetyltransferases or DNA methyltransferases were detected in the available transcriptome data. The gene names and protein lengths, as well as the percentage of identity likeness to the closest species in the databases, are provided in Table 2.

### 4.4. Gene Expression Analysis

#### 4.4.1. RNA Extraction and cDNA Synthesis

RNA extraction and retrotranscription were performed as described in ref. [77] independently for each individual. Briefly, the total RNA was extracted using 500 μL of TRIzol Reagent (Invitrogen, Life Technologies, Carlsbad, CA, USA), following the manufacturer’s protocol. RNA was subsequently treated with 1 μL of DNase I and 5 μL of the corresponding 10× DNase Buffer (Invitrogen, Life Technologies, Carlsbad, CA, USA) and further purified using 200 μL of phenol:chloroform alcohol (Fluka, Neu-Ulm, Germany), using 5PRIME Phase Lock Gel Light tubes (Quantabio, Beverly, MA, USA). Purified RNA was resuspended in nuclease-free water, quantified spectrophotometrically at 260 nm with a BioPhotometer^®^ (Eppendorf, Hamburg, Germany), and stored at −80 °C. For cDNA synthesis, 7.5 μg of RNA was reverse transcribed in a 20 μL reaction using the iScript™ Advanced cDNA Synthesis Kit (Bio-Rad, Hercules, CA, USA) on a C1000 Thermal Cycler (Bio-Rad, Hercules, CA, USA), in accordance with the manufacturer’s instructions.

#### 4.4.2. Real-Time Quantitative PCR

Gene expression analyses were carried out in individuals from *Wolbachia*-infected and uninfected males and females, as indicated in Section 4.1 and Figure 2 and Figure 3. Primers for the selected genes were designed based on the assembled transcriptome, using Primer3 v0.4.0 software [78]. Each of the primer sequences and their corresponding amplicon sizes are provided in Table 3. The size of each qPCR product was confirmed in an agarose gel stained with RedSafe^Tm^ (iNtRON Biotechnology, Kirkland, WA, USA) and visualised using UVIdoc HD2 (Uvitec, Cambridge, UK). Finally, the identities of the amplified fragments were checked using the already-cited BLAST tool. The amplification efficiencies and correlation coefficients for each primer pair were calculated following the Real-Time PCR Applications Guide (Bio-Rad, Hercules, CA, USA catalogue #170–9799). The assay efficiency consistently ranged between 90 and 105% (R^2^ > 0.980).

The gene expression analysis was performed by RT-qPCR under the following conditions: 2 min 50 °C and 2 min initial denaturation at 95 °C, followed by 40 cycles of 15 s denaturation at 95 °C, 30 s annealing and elongation at 60 °C. To ensure the accuracy of each amplicon, a melting curve analysis was conducted after amplification. The identities of amplicons were confirmed by Sanger sequencing (STAB VIDA, Caparica, Portugal). Genes encoding GAPDH and the ribosomal protein L23, which were previously characterised by this group [53], were used as endogenous reference controls to evaluate the relative transcriptional activity of the target genes. The stability of the reference genes was previously evaluated using the BestKeeper and RefFinder programmes [79] among a group of five potential candidates, as already described in ref. [53].

The relative mRNA amount of each target gene was normalised against the expression of the two cited reference genes. A variation in the 2^−ΔΔCt^ Livak method ([80] was employed to analyse the relative changes in gene expression using Design and Analysis app at ThermoFisher© Cloud Dashboard (www.thermofisher.com. Last updated 12 December 2018). Each sample was run in duplicate wells, and two independent technical replicates were performed for each experimental condition. Positive and negative controls were regularly included throughout the assays.

### 4.5. DNA Methylation Analysis

Global DNA methylation of each sample was quantified using MethylFlash Global DNA Methylation (5-mC) ELISA Easy Kit (Colorimetric) (EpiGentek, Farmingdale, NY, USA), following the manufacturer’s instructions. Shortly after, 100 μL of a binding solution were added to each well (of a 96-well plate), followed by 2–4 μL of the DNA sample (samples were diluted to obtain a total of 100 ng of genomic DNA in a volume of 2–4 µL, as suggested by the kit protocol). Positive and negative controls from the kit were included in the plate to generate the standard curve (m = 0.6925; R^2^ = 0.9376). Samples were incubated at 37 °C for 60 min. Later, each well was washed three times with the diluted wash buffer, followed by an incubation with 50 μL of the 5-mC detection complex solution for 50 min. The wells were then rewashed five times, and the samples were incubated for 3 min with 100 μL of colour developer solution. This solution turned blue in the presence of sufficient methylated DNA, while the colour in the negative control wells remained unchanged. When the positive control samples turned blue (indicating the presence of methylated DNA), the enzyme reaction was halted with the stop solution, resulting in a colour change in each sample to yellow. Immediately thereafter, the absorbance (optical density, OD) was read at 450 nm using the Multiskan™ SkyHigh microplate reader (Applied Biosystems, Foster City, CA, USA). With the absorbance results obtained, a 5-mC percentage of each of the samples, from the four experimental categories, was calculated in accordance with the kit protocol.

### 4.6. Data Analyses

The statistical analyses of the gene expression and global DNA methylation results were performed using IBM Statistics SPSS 25.0 (IBM Inc., Armonk, NY, USA), as already described in ref. [53]. The normality and homoscedasticity of data were assessed by means of Shapiro–Wilk and Levene’s tests, respectively, for each parameter. When data were normally distributed and homoscedastic, normalised expression and methylation values were analysed using ANOVA, followed by Bonferroni’s post hoc tests. If the data were normal but not homoscedastic, ANOVA was followed by Games–Howell’s post hoc test. For non-normally distributed data, differences in the gene expression and methylation level were assessed with the non-parametric Kruskal–Wallis test, followed by pairwise comparison and Mann–Whitney–Wilcoxon’s post hoc test. In all cases, *p*-values ≤ 0.05 and ≤0.1 were used as the cut-offs for statistical significance.

## 5. Conclusions

*C. parallelus* offers an exceptional system for studying the influence of *Wolbachia* in non-model species. We have previously shown that this endosymbiotic bacterium alters the transcriptional activity of the genes involved in essential physiological pathways in *Cpp* subspecies, with significantly pronounced effects in females. Here, we have characterised genes related to epigenetic processes for the first time, studied their expression profiles, and analysed the global DNA methylation level of the gonadal tissue of the *Cpp* subspecies, related to *Wolbachia* infection. On one hand, *Wolbachia* modifies the expression of most of these genes, particularly in females, which is relevant given the maternal transmission of this endosymbiont. On the other hand, *Wolbachia* decreases the global DNA methylation level of the gonads of *Cpp* females by 50%. This endosymbiont-induced effect has not been observed in males, but our results show that males show higher DNA methylation in their gonads than females. This preliminary work opens the door to study both the relevance that these variations may have (i) to the *C. parallelus* epigenome and, therefore, to the biology of the grasshopper and the bacterium in their endosymbiotic interaction, and (ii) to the establishment of the CI and reproductive barriers between *C. parallelus* lineages.

## Figures and Tables

**Table 1:** De novo characterised *Cpp* genes. Gene and protein name, ORF and protein lengths of the de novo characterised genes of *Cpp*, as well as their database accession number.

Gene	Protein	ORF Length (pb)	Protein Length (aa)	Accession Number
*H2afy_1*	MacroH2A.1	1146	381	PV661662
*H2A*	H2A	426	141	PV661663
*His2Av*	H2Av	387	128	PV661664
*HDAC1*	HDAC1	1488	495	PV661665
*HDAC3*	HDAC3	1299	432	PV661666
*HDAC6*	HDAC6	3738	1245	PV661667
*HDAC11*	HDAC11	1050	349	PV661668
*SUV39H1*	SUV39H1	2247	748	PV661669
*trx*	TRX	1650	550 ^†^	PV661670
*Gapdh*	Gapdh	999	332	OR824296
*RpL23*	RpL23	423	140	OR824297

^†^ Incomplete protein.

**Table 2:** De novo characterised *C. parallelus* genes related to epigenetic processes. Gene name and protein lengths, as well as the percentage of identity likeness to the closest species on databases, are provided.

Gene	Species	Accession Number	Length (aa)	Identity (%)
*H2afy_1*	*Schistocerca americana*	XP_046982408.1	381	96
	*Schistocerca cancellata*	XP_049764712.1	381	96
*H2A*	*Schistocerca americana*	XP_047001863.1	141	98
	*Schistocerca nitens*	XP_049789131.1	141	97
*His2Av*	*Nasonia vitripennis*	XP_001605065.2	128	100
	*Nilaparvata lugens*	XP_022193185.1	128	100
*HDAC1*	*Schistocerca gregaria*	XP_049860035.1	495	99
	*Schistocerca americana*	XP_046995839.1	495	99
*HDAC3*	*Schistocerca americana*	XP_046979359.1	432	99
	*Zootermopsis nevadensis*	XP_021921778.1	433	95
*HDAC6*	*Schistocerca nitens*	XP_049812413.1	1224	91
	*Schistocerca cancellata*	XP_049780818.1	1224	91
*HDAC11*	*Schistocerca cancellata*	XP_049765096.1	349	97
	*Schistocerca americana*	XP_047003568.1	349	97
*SUV39H1*	*Schistocerca gregaria*	XP_049835843.1	748	84
	*Schistocerca cancellata*	XP_049765228.1	748	84
*trx*	*Schistocerca americana*	XP_046985976.1	3827	79
	*Schistocerca piceifrons*	XP_047103771.1	3870	79
*Gapdh*	*Locusta migratoria*	AFD54028.1	332	99
	*Schistocerca americana*	XP_046988021.1	332	98
*RpL23*	*Zootermopsis nevadensis*	XP_021940908.1	140	100
	*Ischnura elegans*	XP_046404330.1	140	99

**Table 3:** Primers used for cDNA sequencing and RT-qPCR of the genes studied in *Cpp.* Forward (**F**) and reverse (**R**) sequences, and length of amplified fragments.

Gene	Primer Sequence (5′-3′)	Fragment Size (bp)
*H2afy_1*	**F** GACACACCGCTTGCGTATCG **R** GAAGCACTCCTCCTGACGCT	215
*H2A*	**F** TCTGGACGTGGCAAAGGAGG **R** GCCAAGGTAAACGGGAGCAC	153
*His2Av*	**F** CTGGAGTTGGCCGGCAATG **R** AGACTTGTGGATGTGCGGTATCA	153
*HDAC1*	**F** TCAGGCCCGATAACATGTCAGA **R** TGATGCAAACCACCTCCCCA	187
*HDAC3*	**F** GTTACCCGGCAAGATAACGCA **R** GGTCCTCTTCCGCCTGACTC	199
*HDAC6*	**F** TGGCAGCTTCTTTCCTGGGT **R** GCATCAAAGCCCGCAGAGAC	201
*HDAC11*	**F** CCACTGCTGGCTTTGGTGC **R** ACCCACCACCTTTCTGTCCG	169
*SUV39H1*	**F** TGGAGAGGAGTTGACGTTTGACT **R** TGCTTCGCCCCACACTTACA	168
*trx*	**F** AGGATAATGCCGGCGACACA **R** ATCCTCGTCACCTTCGTCACA	242
*Gapdh*	**F** TGCTGATGCCCCAATGTATG **R** TCACGCCACAACTTTCCAGA	219
*RpL23*	**F** TGTCGAAGAGAGGACGAGGTG **R** ATAACCACTGCGGGCATCAC	244

## Data Availability

Data used in this manuscript will be available in the GenBank database (Table 1) as PV661662 (*H2afy_1*), PV661663 (*H2A*), PV661664 (*His2Av*), PV661665 (*HDAC1*), PV661666 (*HDAC3*), PV661667 (*HDAC6*), PV661668 (*HDAC11*), PV661669 (*SUV39H1*) and PV661670 (*trx*).

## Abbreviations

The following abbreviations are used in this manuscript:

5-mC	5-methylcytosine
aa	Amino acids
ANOVA	Analysis of variance
BLAST	Basic Local Alignment Search Tool
bp	Base pairs
cDNA	Complementary deoxiribonucleic acid
CI	Cytoplasmic incompatibility
*Cpp*	*Chorthippus parallelus parallelus*
DNMT	DNA methyltransferase
EDTA	Ethylenediaminetetraacetic acid
EMM	Epigenetic molecular mechanism
F	Forward primer
*H2A*	Histone H2A
*H2afy_1*	Core histone macro-H2A.1 or H2A Histone Family, Member Y
*H2Av*	Histone H2A variant
HAT	Histone acetyltransferases
HDAC	Histone deacetylase
HDM	Histone demethylases
HMT	Histone methyltransferase
KEGG	Kyoto Encyclopedia of Genes and Genomes
mRNA	Messenger ribonucleic acid
NCBI	National Center of Biotechnology Information
ncRNA	Non-coding ribonucleic acid
OD	Optical density
ORF	Open reading frame
qPCR	quantitative PCR
R	Reverse primer
RT	Retrotranscription
*SUV49H1*	Histone-lysine N-methyltransferase SUV39H1
TBE	Tris-Borate-EDTA buffer solution
TE	Tris-EDTA buffer solution
Tm	Melting temperature
*trx*	Histone-lysine N-methyltransferase trithorax
